# Assessing the Relationship between Socioeconomic Status, Race, and Psychological Distress in Cancer Survivors: A Population Based Study

**DOI:** 10.3390/curroncol29040211

**Published:** 2022-04-09

**Authors:** Ikechukwu Chidobem, Fan Tian, Chisom Mgbodile, Francis Mgbodile, Tahereh Orouji Jokar, Esther Ogbuokiri, Nazia Khan

**Affiliations:** 1 Department of Medicine, St. Mary’s General Hospital, Passaic, NJ 07055, USA; chisommgbodile@gmail.com (C.M.); drchykes@gmail.com (F.M.); toroujijokar@primehealthcare.com (T.O.J.); 2 Department of Mathematics, Tufts University, Medford, MA 02155, USA; fan.tian@tufts.edu; 3 Rutgers New Jersey Medical School, Newark, NJ 07103, USA; eo281@njms.rutgers.edu; 4 Atlantic Medical Group Hematology and Oncology, Bridgewater, NJ 08807, USA; nazia.khan@atlantichealth.org

**Keywords:** cancer, psychological distress, race, socioeconomic status

## Abstract

Psychological distress is more common in cancer survivors than the general population, and is associated with adverse outcomes. This cross-sectional study aimed to assess the relationship between socioeconomic status (SES), race and psychological distress, using data from a nationally representative sample of cancer survivors in the United States. Outcomes of interest were mild, moderate, and severe psychological distress as assessed by the Patient Health Questionnaire-4 (PHQ-4). In our univariate model, there was no statistically significant difference in the PHQ-4 scores of Caucasian and African American respondents. On the other hand, a lower SES correlated with a higher likelihood of psychological distress, and this persisted in our multivariate model. This study brings additional awareness to the negative impact of a lower socioeconomic status on mental health outcomes in cancer survivors, and further highlights the importance of the timely identification and screening of individuals at a high risk of psychological distress, in order to limit missed opportunities for relevant mental health interventions in this population.

## 1. Introduction

Socioeconomic status (SES) is generally regarded as a measure of an individual’s or group’s position in society, and is related to exposure to resources, opportunities, and susceptibilities which may influence health [1,2]. Other studies have linked low socioeconomic status to poor health and premature death [3,4]. African Americans (AA) share a disproportionate burden of poverty and have also been shown to have the highest death rate for all cancers combined as well as lower survival rates compared with Caucasian Americans (CA) in the United States (US) [5,6,7]. In order to adequately address healthcare disparities, it is important to consider how race and SES may affect health outcomes [2].

Psychological distress, including depression and anxiety, is more common in cancer survivors than in the general population, and may adversely affect quality of life, treatment, recovery, and survival [8,9,10]. In this study, we sought to assess the relationship between race, SES, and psychological distress using the Patient Health Questionnaire (PHQ-4), which is a screening tool for anxiety and depression. This relationship has been an area of interest for the scientific community; however, it has not been substantially explored. This study aims to add to the existing literature in a bid to provide further illumination in this area, using data from a nationally representative sample of cancer survivors in the US.

## 2. Materials and Methods

### 2.1. Data Source and Study Population

This study used data from the National Cancer Institute’s Health Information National Trends Survey (HINTS). HINTS is a nationally representative survey of noninstitutionalized adults within the US general population aged at least 18 years [11,12]. Data used in this study were obtained from HINTS 5, cycle 4 (the year of data collection for this cycle was 2020). This cycle utilized a two-stage sampling strategy [13].

To achieve the aims of our study, we reported results from the subgroup of CA and AA respondents who self-identified as receiving a cancer diagnosis in the past. This was determined by a “yes” response to the question “have you ever been diagnosed as having cancer”. This comprised 626 individuals (the total number of respondents for this cycle was 3865). From this number, we omitted the cases with missing covariates of interest, which left us with a final study sample of 502 individuals.

### 2.2. Assessing Socioeconomic Status

The markers of SES we used were employment status, insurance coverage, and level of education. Employment status was categorized as full time, retired, student and homemaker; part time; and disabled or unemployed. Insurance coverage was categorized as Medicaid or uninsured; and other non-Medicaid insurances; and unreported. Education level was categorized based on the highest level of completed education as a high-school graduate or less; post-high school or some college; college graduate; and postgraduate.

### 2.3. Assessing Psychological Distress

Psychological distress was assessed using the PHQ-4 questionnaire. The PHQ-4 is an ultrabrief validated screening tool that contains 2 of the core criteria for the diagnosis of depressive disorder and two of the core criteria for the diagnosis of generalized anxiety disorder [14,15]. Based on the total score (range: 0–12), psychological distress is categorized as none (0–2), mild (3–5), moderate (6–9), and severe (10–12) [14,15].

### 2.4. Statistical Analysis

We used descriptive summary statistics to describe demographic and other characteristics of the cohort. Chi-squared tests were used to compare demographical, SES, and PHQ-4 scores across two major racial groups (CA and AA). Multinomial logistic regression models were performed throughout this study to analyze the associations of race and SES measures with the psychological distress level. The odds ratio and 95% confidence interval of the regression models were reported by taking the exponential of the model coefficients and associated standard error. To obtain the corresponding *p*-values, we first computed Z statistics using the ratios of the model coefficients and standard error. Once the Z statistics were computed, we used a two-tailed Wald z-test to test against a significant difference with zero. Final *p*-values were reported at the 0.05 significance level.

Univariate multinomial logistic regression models were performed to analyze the associations between each of the SES measures of employment status, insurance type, and education status with the psychological distress level for the population of CA and AA combined. Furthermore, we performed a multivariate multinomial logistic regression to assess the associations between race and the SES measures with the psychological distress level, while adjusting for age, sex, and BMI. Supplementary analyses were performed with additional adjustment for the smoking status.

All statistical analyses were conducted using R programming (version 4.1.2). Multinomial logistic regression models were performed using the “nnet” package [16].

## 3. Results

Our study cohort consisted of 87.45% (439) CA and 12.55% (63) AA. The mean age of the respondents was 67.12 years (SD: 12.67 years).

Compared to the CA subpopulation, AA respondents in our cohort were slightly older, more male predominant, and had a younger age at cancer diagnosis. AA respondents also had a higher proportion of disabled or unemployed status, higher rates of Medicaid coverage or uninsured status, and a lower education status. The difference in the PHQ-4 scores of both groups did not reach statistical significance in the univariate analysis (Table 1).

Markers of lower socioeconomic status were associated with higher likelihoods of psychological distress in our univariate analysis (Table 2). When using the employment status as the measure of SES, disabled or unemployed respondents had a higher odds ratio for psychological distress (mild: 2.50, 95% confidence interval [CI] 1.16–5.40; moderate: 3.05, 95% CI 0.95–9.75; severe: 11.67, 95% CI 4.24–32.14) compared to other employment statuses. When the insurance status was the measure of interest, participants with Medicaid or no insurance coverage had a higher likelihood of the outcomes of interest (mild: 3.99, 95% CI 2.24–7.13; moderate 3.60, 95% CI 1.41–9.16; severe 10.80, 95% CI 4.16–28.00). When we used the level of education as the SES measure, respondents with graduate degrees had the lowest odds ratio for the outcomes of interest (mild 0.39, 95% CI 0.20–0.77; moderate 0.18, 95% CI 0.05–0.66; severe 0.26, 95% CI 0.05–1.30), followed by respondents with college degrees (mild 0.52, 95% CI 0.28–0.97; moderate 0.40, 95% CI 0.16–1.04; severe 0.37, 95% CI 0.09–1.48).

Compared to CA, AA had a higher odds ratio for the outcomes of interest (mild 1.17, 95% CI 0.60–2.29; moderate 1.71, 95% CI 0.61–4.74; severe 2.61, 95% CI 0.90–7.57).

In our multivariate analysis, lower SES measures were associated with higher likelihoods of psychological distress after adjusting for age, sex, and BMI (Table 3). When using the employment status as the measure of SES, disabled or unemployed respondents had a higher odds ratio for psychological distress (mild: 2.24, 95% CI 1.02–4.93; moderate: 2.21, 95% CI 0.64–7.56; severe: 9.00, 95% CI 3.11–26.05) compared to other employment statuses. When the insurance status was the measure of interest, participants with Medicaid or no insurance coverage had a higher odds ratio for the outcomes of interest (mild: 3.85, 95% CI 2.15–6.90; moderate: 3.09, 95% CI 1.17–8.13; severe: 9.32, 95% CI 3.48–24.99). Among the various education levels, respondents with graduate degrees had the lowest likelihoods of mild and severe psychological distress (mild: 0.38, 95% CI 0.19–0.77; severe: 0.21, 95% CI 0.04–1.08) followed by respondents with college degrees (mild: 0.46, 95% CI 0.24–0.87; severe: 0.22, 95% CI 0.05–0.93).

Compared to CA, AA had a higher odds ratio for the outcomes of interest (mild: 1.23, 95% CI 0.63–2.44; moderate: 1.83, 95% CI 0.63–5.30; severe: 3.65, 95% CI 1.18–11.32).

The above findings were not meaningfully altered by additional adjustments for the smoking status (Table 4).

## 4. Discussion

Our study findings show that lower SES markers correlate with higher risks of psychological distress, as assessed by the PHQ-4, in cancer survivors. Race-based outcomes were similar for both CA and AA in our cohort.

Anxiety and depression are the most common psychological disorders in cancer survivors and negatively impact treatment compliance, quality of life, disease progression, and risk of mortality [17,18,19]. Physical health and mental health have a bi-directional relationship, and it is just as important to screen for affective symptom comorbidity as it is to monitor the physical health of cancer survivors [19]. In our univariate model, disabled or unemployed status as well as Medicaid or uninsured status were all associated with higher likelihoods of having psychological distress. Higher levels of education also correlated with lower likelihoods of having psychological distress, with respondents with graduate degrees having the lowest odds ratio. This is consistent with a multinational European study that showed that a higher education decreased the odds of depression in each country [20]. Other studies have also demonstrated that cancer survivors with less education have more anxiety and higher depressive severity than patients with more education [21,22]. Smoking and a higher BMI are modifiable factors that are usually over-represented in groups with lower SES, and are associated with poorer outcomes in cancer patients [2,23,24]. Adjustment for these confounders did not significantly change our results, suggesting that these modifiers are probably not the only factors that influence outcomes in lower SES groups [2]. In 2014, the American Society of Clinical Oncology (ASCO) released guidelines for depression and anxiety screening in cancer patients [25]. Since 2015, the American College of Surgeons Commission on Cancer (CoC) has also mandated psychosocial distress screening as one of the standards required for the accreditation of cancer centers [26,27]. However, CoC’s standards, which were updated in 2021, only require one screening, with additional screenings left to the discretion of health care providers (HCPs) [27]. Similarly, ASCO guidelines recommend screening for depression and anxiety at the time of the initial cancer visit or diagnosis, and at “appropriate intervals, and as clinically indicated, especially with changes in disease or treatment status (i.e., post-treatment, recurrence, progression) and transition to palliative and end-of-life care” [25]. SES is dynamic throughout life, and cancer survivors have been shown to be more likely to lose employment and less likely to regain employment than cancer-free individuals [2,28]. Therefore, it is imperative that HCPs be able to recognize cancer survivors at risk of anxiety or depression, as indicated by a downward change in their SES, and promptly re-screen those whose initial screening was negative and who do not otherwise meet criteria for re-screening based on ASCO’s specification of “changes in disease or treatment status” [25].

In the multivariate analysis, Medicaid or uninsured status as well as disabled or unemployed status all correlated with higher likelihoods of psychological distress. Respondents with graduate degrees had the least likelihood of having mild and severe psychological distress after adjusting for covariates. There was no statistically significant difference in psychological outcomes between CA and AA. This suggests that lower SES is associated with similar psychological outcomes in CA and AA, thus highlighting the importance of considering the needs of all cancer survivors, rather than just their race [29]. To add to this point, there was no statistically significant difference in the PHQ-4 scores of AA and CA in our univariate analysis. In a recent US-based study, there were no race-based differences in psychological distress between CA and AA cancer survivors after adjusting for covariates [29]. Another study in cancer survivors found that SES, but not minority status, was associated with depressive severity [21]. The attenuating effect of education on the risk of adverse mental health has been shown in other studies and may be due to an association between more education and greater coping mechanisms, an increased practice of positive health habits, and better access to adequate healthcare, including preventive measures [21,22,30,31]. Since information about socioeconomic status is easy to obtain in healthcare settings, this could be an area in which cancer programs could develop intervention strategies to improve mental health outcomes in cancer survivors [31]. One such strategy is the facilitation of contact with mental health professionals who are trained to deal with cancer survivors. In addition, HCPs could also spend additional time ensuring that cancer survivors with a low socioeconomic status understand the possible negative impact of poor mental health on outcomes, and provide these individuals with community resources such as support groups. This is especially important as cancer survivors may be hesitant to discuss issues related to mental health with their HCPs and may instead focus more attention on therapies that are directly connected to cancer-specific mortality reduction, because they believe cancer is their greatest threat to survival [32]. Our study had some limitations. First, the diagnosis of cancer was self-reported and not corroborated with medical records. Another limitation is the potential for non-response bias, since cancer survivors who answered the survey may be different from nonresponders, and this may reduce the generalizability of the study results [33]. Additionally, our study did not make a distinction between cancer types, time from diagnosis, or prognosis. It will be interesting to see how these factors influence psychological distress in cancer survivors. Additionally, CA made up 87% of the sample. This may indicate that our study is more powered to find associations in CA compared to AA. [2] Additionally, although there is no consensus on the measures that influence SES, it would also be useful for further studies to include additional SES markers such as income and residence in order to create a more holistic measurement for SES [1,2,20]. Other limitations include possible unidentified confounders, as well as the cross-sectional nature of the HINTS survey [2]. Given the dynamic nature of SES, longitudinal studies will be helpful in evaluating how risks of psychological distress in cancer survivors are affected by changes in their SES, as this will lend further support to an association between these factors.

## 5. Conclusions

Our study demonstrated a correlation between a lower socioeconomic status and higher likelihood of psychological distress in cancer survivors. There was no statistically significant difference in the outcomes of interest among AA and CA respondents. This study adds to the present literature regarding the relationship between SES, race, and psychological distress in this population. Prompt identification and screening of patients at a higher risk of psychological distress will limit missed opportunities for mental health interventions in cancer survivors and help reduce existing health disparities.

## Figures and Tables

**Table 1:** Baseline characteristics of participants.

Characteristic	Whole Sample 502	Caucasian/White 439	African American/Black 63	*p* Value
Age, mean ± SD; y	67.12 ± 12.67	66.89 ± 13	68.71 ± 9.94	**<0.001**
Female, *n* (%)	290 (57.77%)	258 (58.77%)	32 (50.79%)	**0.232**
Race, *n* (%)				
Caucasian/White	439 (87.45%)	439 (100%)	-	
African American/Black	63 (12.55%)	-	63 (100%)	
Age at diagnosis, mean ± SD; y	53.56 ± 15.79	53.75 ± 15.51	52.18 ± 17.73	**0.473**
Body Mass Index (BMI), mean ± SD; kg/m^2^	28.49 ± 6.12	28.27 ± 5.99	30.03 ± 6.84	**0.0324**
Smoking status, *n* (%)				**0.586**
Never	266 (52.99%)	232 (52.85%)	34 (53.97%)	
Former	177 (35.26%)	153 (34.85%)	24 (38.1%)	
Current	59 (11.75%)	54 (12.3%)	5 (7.94%)	
Employment status, *n* (%)				**<0.001**
Group 1				
Full time	147 (29.28%)	141 (32.12%)	6 (9.52%)	
Retired	259 (51.59%)	226 (51.48%)	33 (52.38%)	
Homemaker	28 (5.58%)	24 (5.47%)	4 (6.35%)	
Student only	1 (0.2%)	1 (0.23%)	0 (0%)	
Group 2:				
Part time	25 (4.98%)	21 (4.78%)	4 (6.35%)	
Group 3:				
Disabled	33 (6.57%)	22 (5.01%)	11 (17.46%)	
Unemployed	9 (1.79%)	4 (0.91%)	5 (7.94%)	
Insurance, *n* (%)				**<0.001**
Medicaid/Uninsured	74 (14.74%)	55 (12.53%)	19 (30.16%)	
Other	428 (85.26%)	384 (87.47%)	44 (69.84%)	
Education, *n* (%)				**0.0151**
High school or lower	133 (26.49%)	106 (24.15%)	27 (42.86%)	
Post-high school or some college	147 (29.28%)	131 (29.84%)	16 (25.4%)	
College graduate	119 (23.71%)	107 (24.37%)	12 (19.05%)	
Postgraduate	103 (20.52%)	95 (21.64%)	8 (12.7%)	
PHQ-4 Total Score				**0.254**
Normal (0–2)	354 (70.52%)	314 (71.53%)	40 (63.49%)	
Mild psychological distress	100 (19.92%)	87 (19.82%)	13 (20.63%)	
Moderate psychological distress (6–8)	28 (5.58%)	23 (5.24%)	5 (7.94%)	
Severe psychological distress (9–12)	20 (3.98%)	15 (3.42%)	5 (7.94%)	

**Table 2:** Univariate model (CA & AA). PHQ-4 score change and 95th percentile confidence interval for correlations between socioeconomic status and PHQ-4 score.

PHQ-4	Normal	Mild		Moderate		Severe	*p*-Value
		Odds Ratio (95% CI)	*p*-Value	Odds Ratio (95% CI)	*p*-Value	Odds Ratio (95% CI)	
Employment Status							
Full time, retired, student and homemaker	Reference						
Part time		0.75 (0.25, 2.26)	0.609	0.69 (0.09, 5.34)	0.72	0 (0, 1.13 ×10^16^)	0.787
Disabled, unemployed		2.50 (1.16, 5.40)	0.019	3.05 (0.95, 9.75)	0.06	11.67 (4.24, 32.14)	<0.001
Insurance Status							
Other	Reference						
Medicaid/Uninsured		3.99 (2.24, 7.13)	<0.001	3.60 (1.41, 9.16)	0.007	10.80 (4.16, 28.00)	<0.001
Education							
High school or lower	Reference						
Post-high school or some college		0.59 (0.33, 1.04)	0.070	0.14 (0.04, 0.51)	0.003	0.81 (0.28, 2.33)	0.699
College graduate		0.52 (0.28, 0.97)	0.038	0.40 (0.16, 1.04)	0.060	0.37 (0.09, 1.48)	0.160
Postgraduate		0.39 (0.20, 0.77)	0.007	0.18 (0.05, 0.66)	0.009	0.26 (0.05, 1.30)	0.101
Race							
Caucasian/White	Reference						
African American/Black		1.17 (0.60, 2.29)	0.64	1.71 (0.61, 4.74)	0.305	2.61 (0.90, 7.57)	0.077

**Table 3:** Multivariate model. PHQ-4 score change and 95th percentile confidence interval for adjusted associations between socioeconomic status, race, and PHQ-4 score. All values were adjusted for age, sex, and BMI.

PHQ-4	Normal	Mild		Moderate		Severe	*p*-Value
		Odds Ratio (95% CI)	*p*-Value	Odds Ratio (95% CI)	*p*-Value	Odds Ratio (95% CI)	
Employment Status							
Full time, retired, student and homemaker	Reference						
Part time		0.7 (0.23, 2.14)	0.534	0.58 (0.07, 4.69)	0.609	0 (0, 0)	<0.001
Disabled, unemployed		2.24 (1.02, 4.93)	0.045	2.21 (0.64, 7.56)	0.208	9.00 (3.11, 26.05)	<0.001
Insurance Status							
Other	Reference						
Medicaid/Uninsured		3.85 (2.15, 6.90)	<0.001	3.09 (1.17, 8.13)	0.022	9.32 (3.48, 24.99)	<0.001
Education							
High school or lower	Reference						
Post-high school or some college		0.55 (0.31, 0.99)	0.047	0.13 (0.04, 0.48)	0.002	0.65 (0.22, 1.95)	0.442
College graduate		0.46 (0.24, 0.87)	0.018	0.36 (0.13, 0.97)	0.043	0.22 (0.05, 0.93)	0.040
Postgraduate		0.38 (0.19, 0.77)	0.007	0.20 (0.05, 0.75)	0.017	0.21 (0.04, 1.08)	0.061
Race							
Caucasian/White							
African American/Black		1.23 (0.63, 2.44)	0.543	1.83 (0.63, 5.30)	0.263	3.65 (1.18, 11.32)	0.025

**Table 4:** Multivariate model. PHQ-4 score change and 95th percentile confidence interval for adjusted associations between socioeconomic status, race, and PHQ-4 score. All values were adjusted for age, sex, BMI, and smoking status.

PHQ-4	Normal	Mild		Moderate		Severe	*p*-Value
		Odds Ratio (95% CI)	*p*-Value	Odds Ratio (95% CI)	*p*-Value	Odds Ratio (95% CI)	
Employment Status							
Full time, retired, student and homemaker	Reference						
Part time		0.65 (0.21, 2)	0.451	0.57 (0.07, 4.63)	0.599	0 (0, 0)	<0.001
Disabled, unemployed		2.11 (0.95, 4.67)	0.066	2.1 (0.6, 7.3)	0.242	7.15 (2.39, 21.42)	<0.001
Insurance Status							
Other	Reference						
Medicaid/Uninsured		3.73 (2.07, 6.71)	<0.001	3.08 (1.16, 8.21)	0.025	8.66 (3.17, 23.66)	<0.001
Education							
High school or lower	Reference						
Post-high school		0.55 (0.31, 0.99)	0.047	0.13 (0.04, 0.49)	0.002	0.67 (0.22, 2.06)	0.481
College		0.47 (0.25, 0.9)	0.024	0.37 (0.13, 1.01)	0.052	0.25 (0.06, 1.1)	0.066
Graduate		0.4 (0.2, 0.81)	0.010	0.2 (0.05, 0.76)	0.018	0.27 (0.05, 1.45)	0.126
Race							
Caucasian/White	Reference						
African American/Black		1.26 (0.64, 2.49)	0.509	1.78 (0.61, 5.18)	0.287	4.76 (1.45, 15.64)	0.010

## Data Availability

The data used in this study are available, upon request, from the United States National Cancer Institute.
